# RNA m^6^A methylation in cancer

**DOI:** 10.1002/1878-0261.13326

**Published:** 2022-11-06

**Authors:** Zhaotong Wang, Jiawang Zhou, Haisheng Zhang, Lichen Ge, Jiexin Li, Hongsheng Wang

**Affiliations:** ^1^ School of Pharmaceutical Sciences Sun Yat‐sen University Guangzhou China

**Keywords:** cancers, m^6^A, m^6^A modulators, mRNA, ncRNA, therapy

## Abstract

*N* ^6^ *‐*methyladenosine (m^6^A) is one of the most abundant internal modifications in eukaryotic messenger RNAs (mRNAs) and non‐coding RNAs (ncRNAs). It is a reversible and dynamic RNA modification that has been observed in both internal coding segments and untranslated regions. Studies indicate that m^6^A modifications play important roles in translation, RNA splicing, export, degradation and ncRNA processing control. In this review, we focus on the profiles and biological functions of RNA m^6^A methylation on both mRNAs and ncRNAs. The dynamic modification of m^6^A and its potential roles in cancer development are discussed. Moreover, we discuss the possibility of m^6^A modifications serving as potential biomarkers for cancer diagnosis and targets for therapy.

## Graphical abstract

N6‐methyladenosine (m^6^A) is a widespread regulator not only in messenger RNAs but also in non‐coding RNAs. m^6^A can be reversibly methylated and unmethylated by “m^6^A writer” and “m^6^A eraser” proteins. The methylated RNA is recognized by m^6^A “readers” protein and then to modulate cancer progression. Target m^6^A regulators has been becoming a promising approach for cancer diagnosis and treatment.

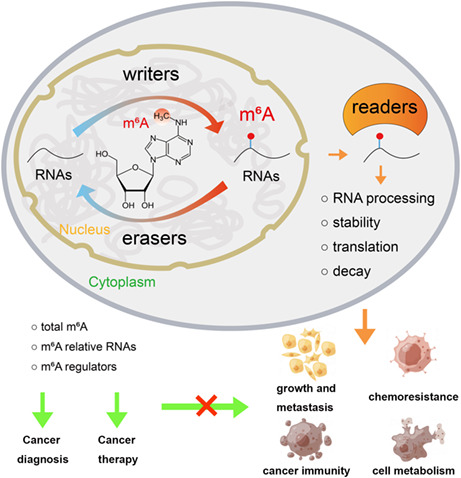

**Abbreviations**
ALKBH: AlkB homolog
AML: acute myeloid leukemia
APA: alternative polyadenylation
BC: breast cancer
BCA: bladder cancer
carRNA: chromosome‐associated regulatory RNA
CDS: protein‐coding sequence
ceRNA: competitive endogenous RNA
circRNA: circular RNA
CRC: colorectal cancer
eIF3: eukaryotic initiation factor 3
EMT: epithelial–mesenchymal transition
EOC: endometrioid cancer
eRNA: enhancer RNA
ESCC: esophageal squamous cell carcinoma
FTO: obesity‐associated protein
GBM: glioblastoma
GC: gastric cancer
HCC: hepatocellular carcinoma
HDGF: hepatoma‐derived growth factor
hESCs: human embryonic stem cells
HMGA: high mobility group protein
HNRNPs: heterogeneous nuclear ribonucleoproteins
HMGA2: high mobility group protein 2
IGF2BPs: insulin‐like growth factor 2 mRNA‐binding proteins
KIAA1429/virilizer: virilizer like m^6^A methyltransferase associated protein
LC: lung cancer
lncRNA: long non‐coding RNA
lincRNA: long intergenic non‐coding RNA
LUAD: lung adenocarcinoma
m^6^A: N6‐methyladenosine
METTL3: methyltransferase‐like 3
MTase: methyltransferase
MTC: methyltransferase complex
NPC: nasopharyngeal carcinoma
NSCLC: non–small cell lung cancer
OC: ovarian cancer
OS: osteosarcoma
paRNA: promoter‐associated RNA
PAAD: pancreatic adenocarcinoma
PPP: pentose phosphate pathway
PRAD: prostate cancer
Prrc2a: proline‐rich coiled‐coil 2 A
RB: retinoblastoma
RBM: RNA‐binding motif
RBP: RNA‐binding protein
RCC: renal cell carcinoma
RNAP II: RNA polymerase II
R‐2HG: R‐2‐hydroxyglutarate
rRNA: ribosomal RNA
SAM: S‐adenosylmethionine
SJ: splice junction
SRSF3: serine and arginine‐rich splicing factor 3
TC: thyroid cancer
tRNA: transfer RNA
UTR: untranslated terminal region
WTAP: Wilms tumor 1–associated protein
XIST: X‐inactive specific transcript
YTH: YT521‐B homology
ZCCHC4: CCHC zinc finger‐containing protein
ZC3H13: zinc finger CCCH‐type containing 13

## 1 Introduction

**Fig. 1:**
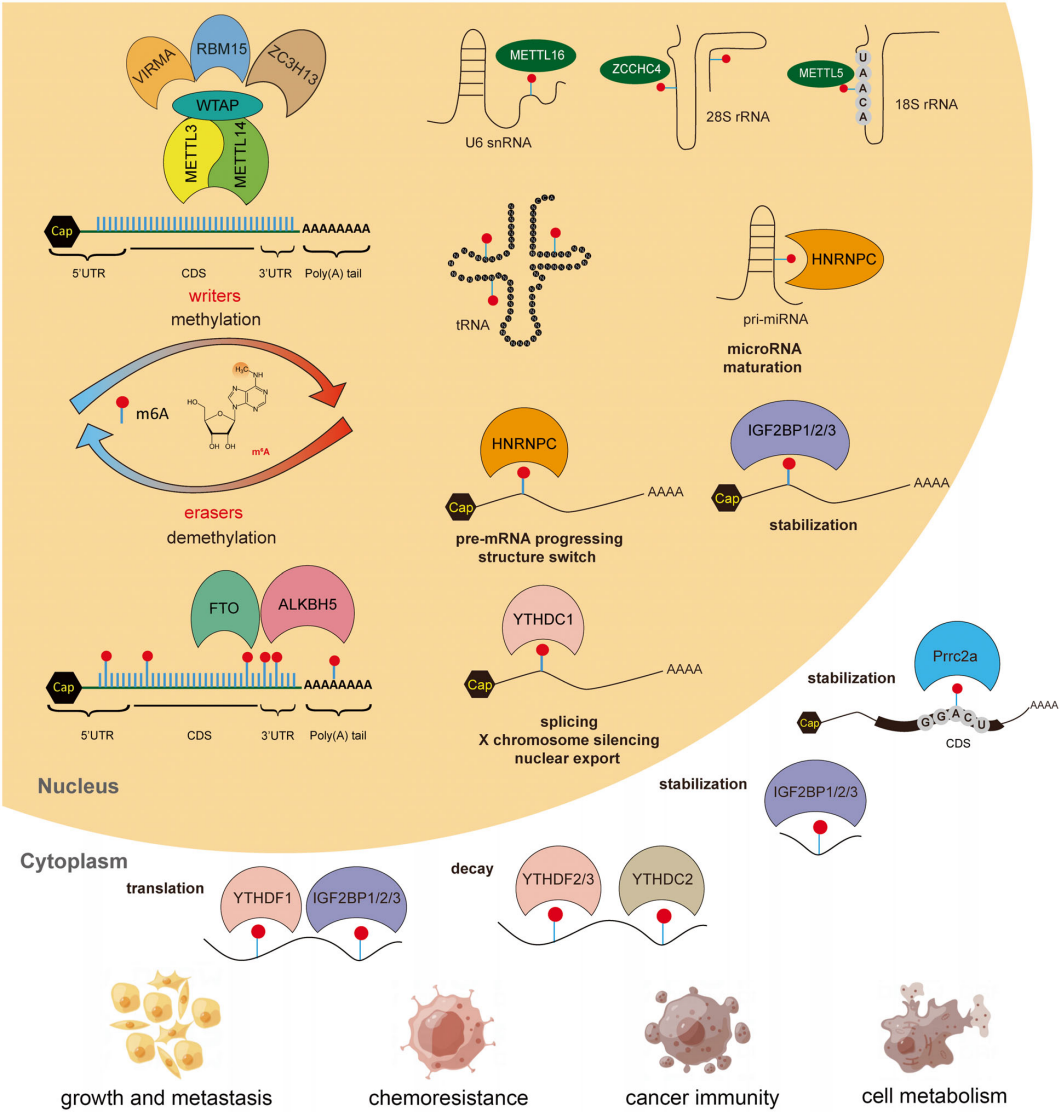
Molecular reaction for m^6^A methylation and its functions in cancer development. m^6^A writers (METTL3, METTL14, WTAP, VIRMA, RBM15, ZC3H13, METTL5, METTL16 and ZCCHC4) and m^6^A erasers (FTO and ALKBH5) mediate the m^6^A methylation/demethylation of RNAs, including mRNA, tRNA, rRNA, snRNA and pre‐miRNA. m^6^A readers (YTHDF1‐3, YTHDC1‐2, HNRNPs, IGF2BP1‐3 and eIF3), locating in either nucleus or cytoplasm, bind to RNA targets and play different roles in the regulation of RNA behaviors such as RNA processing and decay. All m^6^A modulators are involved in cancer growth and metastasis, cancer chemoresistance, cancer immunity and cell metabolism [3, 4, 5, 6, 7, 8, 9, 10, 11, 12, 13, 14, 15, 16, 17, 18, 19, 20].

RNA modifications were discovered more than 50 years ago, and over 170 chemical modifications on RNA have so far been identified [1]. *N* ^6^‐methyladenosine (m^6^A) is the most prevalent internal modification on eukaryotic RNAs including messenger RNA (mRNA) and non‐coding RNA (ncRNA). The N6 position of adenosine can be reversibly methylated and unmethylated by ‘m^6^A writer’ and ‘m^6^A eraser’ proteins, respectively, and m^6^A RNA can be recognized and bound by ‘m^6^A reader’ proteins [2] (Fig. 1, Table 1, Box 1).

**Table 1:** m^6^A writers, erasers and readers and their functions in cancers.

Type	Protein	Role/effect	
		Promote	Suppress
Writer	METTL3	*Cancer progression* CRC: Stabilization of *HK2* and *SLC2A1* mRNAs [21] BC: Feedback loop of HBXIP/let‐7g/METTL3/HBXIP [22] ESCA: Stabilization of *Notch* mRNA [23] *Cell differentiation and cell proliferation* AML: Promotion of the translation of c‐*MYC*, *BCL2* and *PTEN* mRNAs [24] *Glycolysis and tumorigenesis* CESC: Promotion of the translation of *PDK4* mRNA [25] LUAD: Promotion of the translation of *ENO1* mRNA [26]	*Tumor metastasis* TNBC: Down‐regulation of *COL3A1* mRNA [27]
	METTL14	*Leukemogenesis* AML: Regulation of *MYB* and *MYC* mRNA [28] *Cancer progression* BRCA: Stabilization of *CXCR4* and *CYP1B1* mRNAs [29] ESCA: Decreased expression of PHLPP2 and increased expression of mTORC2 [30] *Tumor metastasis* PRAD: Increased of *PERP* mRNA turnover [31] *Tumor malignancy* CESC: Stabilization of *CYP1B1* mRNA [32]	*Tumor metastasis* PRAD: Degradation of *SOX4* mRNA [33] *Tumor malignancy* HCC: Degradation of *USP48* mRNA [34] *Cell self‐renewal and tumorigenesis* BCA: Degradation of *Notch1* mRNA [35]
	METTL16	*Cell proliferation* GC: Stabilization of *CCND1* mRNA [36] *Translation and tumorigenesis* Promotion of the translation of over 4000 mRNA transcripts [37]	
	METTL5	*Cancer progression* PRAD: Modulation of the translation of *c‐Myc* mRNA [38] *Cell proliferation* BC: Promotion of translation initiation [39]	
	WTAP	*Cancer progression* HCC: Post‐transcriptional suppression of *ETS1* mRNA [40]	
	KIAA1429	*Cancer progression* HCC: Reduced the interaction between *HuR* and *MMP1* mRNAs [41]	
	RBM15	*Cancer progression* LSCC: Stabilization of *TMBIM6* mRNA [42]	
	ZC3H13		*Cell proliferation and invasion* CRC: Inactivation of Ras‐ERK signaling [43]
Eraser	FTO	Leukemogenesis AML: Degradation of *ASB2* and *RARA* mRNA [44] *Cancer progression* BRCA: Degradation of *BNIP3* mRNA [45]	*Stem cell self‐renewal* OC: Degradation of *PDE1C* and *PDE4B* mRNAs [46] *Tumor metastasis* CRC: Degradation of *MTA1* mRNA [47]
	ALKBH5	*Tumorigenesis* MM: Stabilization of *TRAF1* mRNA [48] *Cancer progression* GBM: Enhance expression of *FOXM1* mRNA [49]	*Tumorigenesis* PDAC: Enhance expression of *WIF‐1* mRNA and mediation of the Wnt pathway [50]
Reader	YTHDF1	*Tumorigenesis* CRC: Enhanced the translation of *ARHGEF2* mRNA [51] GC: Promotion of the translation of *FZD7* mRNA [52] HCC: Promotion of the translation of *TRFC* mRNA [53] *Cancer progression* OC: Promotion of the translation of *EIF3C* mRNA [54]	
	YTHDF2	*Stem cell self‐renewal* AML: Degradation of *TNFRSF2* mRNA [55] *Cancer progression* GBM: Stabilization of *MYC* and *VEGFA* mRNAs [56] *Tumorigenesis* OM: Degradation of *PER1* and *TP53* mRNAs [57]	*Cell proliferations* PRAD: Stabilization of *YAP* mRNA and regulation of TGF‐β/Smad signaling [58]
	YTHDF3	*Tumorigenesis* OM: Promotion of the translation of *CTNNB1* mRNA [59] *Tumor metastasis* BC: Promotion of the translation of *ST6GALNAC5*, *GJA1*, and *EGFR* mRNAs [60]	
	YTHDC1	*Cell proliferations* AML: Stabilization of *MCM4* mRNA and regulation of DNA replication [61]	*Tumorigenesis* PRAD: Stabilization of mature miR‐30d and inhibition of aerobic glycolysis [62]
	YTHDC2	*Cancer progression* GC: Promotion of the translation of *YAP* mRNA [63] Tumor metastasis Promotion of the translation of *HIF‐1α* mRNA [64]	*Tumorigenesis* LUAD: Degradation of *SLC7A11* mRNA [65]
	IGF2BP1	*Cancer progression* EC: Stabilization of *PEG10* mRNA [66] *Stem cell stemness* BC: Stabilization of *c‐Myc* mRNA [67] *Tumor metastasis* BC: Stabilization of *KRT7‐AS/KRT7* mRNA duplex [68]	*Cancer progression* BCA: Degradation of *MYC* and *FSCN1* mRNAs [69]
	IGF2BP2	*Cell proliferation* CRC: Stabilization of *HMGA1* mRNA [70] HCC: Stabilization of *FEN1* mRNA [71] PRAD: Activation the PI3K/Akt signaling pathway [72] *Tumor metastasis* PRAD: Stabilization of *IGF1R* mRNA [73]	
	IGF2BP3	*Cell proliferation* BCA: Activation of the JAK/STAT pathway [74] *Angiogenesis* CRC: Degradation of *CCND1* mRNA [75] *Tumor metastasis* PRAD: Stabilization of *HDAC4* mRNA [76]	
	hnRNPR	*Cell proliferation and metastasis* GC: Stabilization of *CCNB1* and *CENPF* mRNAs [77, 78, 79, 80, 81]	*Cancer progression* BCA: Mediation of *PKM* alternative splicing [82]

Box 1 **RNAs and m^6^A‐related proteins**

**rRNA:** ribosomal ribonucleic acid is the component of ribosomes to process protein synthesis. **lncRNAs**: are longer than 200 nucleotides that do not encode proteins, including both intergenic and genic non‐coding RNA. **lincRNA:** long intergenic non‐coding RNAs are longer than 200 nucleotides which constitute more than half of lncRNA transcripts in humans. LincRNAs are non‐coding RNA transcripts that make up most of the lncRNAs. **miRNA:** is a 21‐25nt single‐stranded non‐coding RNA. It plays a role in RNA silencing and post‐transcriptional regulation of gene expression. **paRNA:** promoter‐associated RNAs is a type of lncRNA, which could influence promoter activity of other genes. **eRNA:** enhancer RNA is a type of lncRNA transcribed from the DNA sequence of enhancer regions. **circRNA:** is a type of single‐stranded RNA formed into continuous loop. It also shows potential to code for proteins. **m** ^**6**^ **A writer:** is a methyltransferase complex (MTC), which catalyzes m^6^A deposition through transferring a methyl group from donor S‐adenosylmethionine (SAM) and includes METTL3, METTL14, WTAP, METTL16, METTL5, KIAA1429/Virilizer, RBM15, ZCCHC4 and ZC3H13; **m** ^**6**^ **A eraser:** is a demethylase which reverts m^6^A to adenosine on RNAs, including FTO and ALKBH5; **m** ^**6**^ **A reader:** is executer to exert functions of m^6^A and plays important roles in epigenetics, including YTH family proteins, HNRNPs, IGF2BPs, eIF3 and Prrc2a.

### 1.1 m^6^A writers, erasers and readers

The known m^6^A writers include METTL3 [3], METTL14 [3], WTAP [3], METTL16 [4], METTL5 [5], KIAA1429/Virilizer [6], RBM15 [6], ZCCHC4 [7] and ZC3H13 [8]. An m^6^A ‘writer’ is an MTase complex (MTC), which catalyzes m^6^A deposition by transferring a methyl group from donor S‐adenosylmethionine (SAM) [3]. METTL3 is a 70 kDa protein highly conserved in eukaryotic cells belonging to class I MTases, which contains a conserved SAM‐binding domain [3] to recognize the DRACH motif of RNA, whose consensus sequence is D = A/G/U, R = A/G and H = A/C/U [83]. METTL14 forms a heterodimer with METTL3, facilitating METTL3 binding with target RNA in MTC [3]. WTAP is indispensable to the MTC by binding with the N‐terminal helix of METTL3, acting as a regulatory subunit of MTC [84]. In the absence of WTAP, the RNA binding ability of the MTC is highly reduced [84]. KIAA1429, also known as VIRMA, tends to bind the 3′UTR, near mRNA stop codons, recruiting MTC to enhance region‐selective m^6^A methylation [6]. RBM15/15B, interacts with METTL3 in a WTAP‐dependent manner to support m^6^A modification and promote RNA splicing (Box 2) [6, 85]. ZC3H13 is required for the nuclear localization of the ZC3H13‐WTAP‐Virilizer‐Hakai complex to facilitate m^6^A methylation in 3′UTR of targets [8]. METTL5 is a newly discovered m^6^A writer of 18S ribosomal RNA (rRNA; Box 1), binding to a UAACA motif and catalyzing m^6^A 1832 in 18S rRNA [5]. METTL16 catalyzes m^6^A methylation on U6 spliceosomal snRNA, which is associated with the expression of SAM synthetase [4]; ZCCHC4 deposits m^6^A on a subset of mRNAs as well as 28S rRNA [7].

Box 2 **Functional consequences of m^6^A modification on mRNA**

**Fig. 2:**
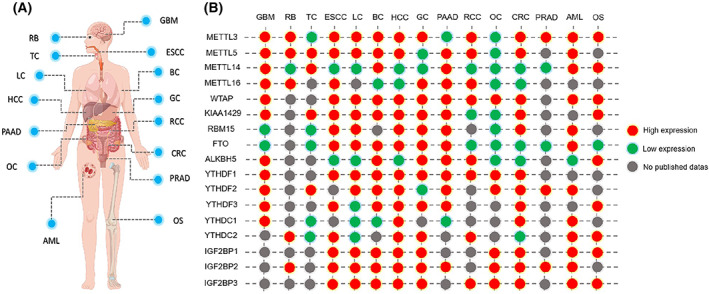
Functions of m^6^A modification on mRNA. A schematic image of the roles of m^6^A on mRNA. m^6^A modification on mRNA plays different roles in nucleus and cytoplasm. (A) Regulation of transcription, (B) regulation of splicing, (C) alteration of RNA structure, (D) facilitation of mRNA export, (E) determination of APA, (F) regulation of translation and (G) regulation of mRNA stability.

**mRNA transcription:** mRNA transcription can be regulated by chromosome‐associated regulatory RNAs (carRNAs). carRNAs can be modified by m^6^A methylation. Reduction of m^6^A in selected carRNAs elevates carRNAs levels and promotes an open chromatin state and downstream transcription [86]. Moreover, YTHDC1 recruits the H3K9me2 demethylase KDM3B to m^6^A‐associated chromatin region, where H3K9me2 demethylation initiates gene expression [87]. Finally, m^6^A methyltransferase complex promotes RNAP II pause release and affects nascent RNA transcription [88] (Fig. 2A).

**Splicing:** m^6^A participates in pre‐mRNA processing and regulation of alternative splicing [89]. Early m^6^A was deposited near the splice junctions (SJs) and introns of nascent RNA, whilst these signals disappeared in mature RNAs [90]. Early co‐transcriptional m^6^A deposition near SJs promotes fast splicing, and the presence of m^6^A modifications in introns is associated with long, slowly processed introns and alternative splicing events. In addition, YTHDC1 can recognize m^6^A on alternative exons, which recruits the splicing factor serine and arginine‐rich splicing factor 3 (SRSF3) but restricts binding with exon‐skipping factor SRSF10, resulting in exon inclusion during alternative splicing [14] (Fig. 2B).

**mRNA structure:** RNA secondary structure is formed by nucleotide bases paired within its sequence via hydrogen bonding, forming the scaffold and the folding of RNA three‐dimensional structures [91]. m^6^A can weaken the A/U pairings, leading to the alterations of RNA secondary structure and thermostability of RNA duplexes. These structural changes would influence the interaction of related regulatory proteins, such as hnRNP and HNRNPs, leading to the inhibition of RNA‐protein interactions [16] (Fig. 2C).

**mRNA export:** m^6^A might act as export signals for mRNAs. Treatment with methylase inhibitor S‐tubercidinylhomocysteine reduces m^6^A level and attenuates mRNA export [92]. ALKBH5 knockdown leads to m^6^A‐modified mRNA accumulation in cytoplasm [93], whereas YTHDC1 knockdown extends residence time for nuclear m^6^A‐containing mRNAs, with an accumulation of transcripts in the nucleus and accompanying depletion within the cytoplasm [94] (Fig. 2D).

**Alternative polyadenylation (APA):** APA is an important post‐transcriptional regulation mechanism that targets the 3′end of pre‐mRNA during mRNA maturation in eukaryotic cells. As a result of APA, there are multiple transcripts for over half of human genes [95]. Bioinformatic analysis suggests a possible connection of m^6^A to polyA site choices in mRNA: m^6^A is preferentially located within 3′UTRs containing multiple APA and regulates proximal APA choice [96]. As APA regulates the stability, translation and location of mRNAs, m^6^A might also regulate mRNA behaviors indirectly via modulation of APA choice (Fig. 2E).

**Translation:** m^6^A modulates translational dynamics by potentially influencing the progress of different stages. 5′UTR m^6^A promotes cap‐independent translation by directly binding to eIF3 [97]; CDS m^6^A acts as a barrier to tRNA accommodation to regulate translation‐elongation dynamics [98]; 3′UTR m^6^A facilitates the translation by METTL3‐eIF3h‐mediated mRNA circularization [99]. m^6^A might also play roles in both translation initiation and elongation: CDS m^6^A can enhance mRNA translation by relieving ribosome stalling [100] or trigger polysome‐mediated translation in the case of Snail mRNA [101]; Conversely, decrease m^6^A promoted eIF4E3‐mediated cap‐independent translation of β‐catenin [102]. Whilst m^6^A deposition in transcripts may regulate mRNA translation, a complete picture of how translation is regulated is currently lacking (Fig. 2F).

**mRNA stability:** m^6^A modification has been shown to regulate mRNA stability, dependent on its bound m^6^A readers. m^6^A‐containing mRNAs underwent two distinct pathways of rapid degradation: deadenylation via YTHDF2‐CCR4/NOT (deadenylase) complex or YTHDF2‐HRSP12‐RNase P/MRP (endoribonuclease) complex [103]. m^6^A‐modified mRNAs can also be targeted toward an opposite fate. For instance, IGF2BP proteins can increase the half‐lives of m^6^A‐containing mRNAs [17] (Fig. 2G).

Conversely, ‘erasers’ revert m^6^A to adenosine on RNAs. The identified m^6^A erasers are fat mass and obesity‐associated protein (FTO) [104] and AlkB Homolog 5 (ALKBH5) [93]. Both FTO and ALKBH5 require ferrum and α‐ketoglutaric acid as co‐factors to remove m^6^A in eukaryotic cells [105]. However, they demethylate different targets due to their different structural interaction. FTO contains a C‐terminal domain which is easy to engage in protein–RNA interaction, while the isolated N‐terminal domain is incompetent for catalysis [106]. Regarding m^6^A in mRNA, cap m^6^Am, m^1^A and m^6^Am in snRNA are the substrates of FTO *in vivo* [9]. ALKBH5, which is predominant in the nucleus, can directly bind to RNA substrates and be a part of the mRNA‐bound proteome [93, 107].

‘m^6^A readers’ are executers to exert functions of m^6^A and play important roles in epigenetics, including YTH family proteins, HNRNPs, IGF2BPs, eIF3 and Prrc2a [108]. Among them, YTH family proteins are the most studied m^6^A readers, including YTHDF1, YTHDF2, YTHDF3, YTHDC1 and YTHDC2 [109]. Among them, YTHDF1‐3 paralogs have been reported to mediate the major effects of m^6^A on RNA regulations [110]: YTHDF1 enhances mRNA translation [10]; YTHDF2 accelerates the decay of m^6^A‐modified transcripts [11]; YTHDF3 enhances both mRNA translation and degradation (Box 2, Fig. 2) [12]. Aside from YTH conserved domain, YTHDC1 and YTHDC2 are not related to paralogs proteins and play different roles in cells [13]: YTHDC1 is the only known m^6^A reader in the nucleus, regulating RNA splicing and translocation [14], while YTHDC2 enhances translation of target RNAs by recruiting other protein complexes [15]. Additional m^6^A reader proteins have been identified such as the HNRNP family containing hnRNPA2/B1, HNRNPC and HNRNPF involved in promoting primary microRNA processing [111], mRNA alternative splicing, processing of target transcripts and interaction of m^6^A‐rich long non‐coding RNA (lncRNA; Box 1) [16]. m^6^A readers in the IGF2BPs family include IGF2BP1, IGF2BP2 and IGF2BP3. The binding of m^6^A‐methylated mRNA with IGF2BPs protein resulted in the up‐regulation of mRNA stability (Box 2) [17]. Recently, Prrc2a was identified as a novel m^6^A reader binding to GGACU motif in the CDS region of mRNAs via an m^6^A‐dependent manner, which then stabilized m^6^A‐modified mRNAs [18].

### 1.2 m^6^A profiles of RNAs

m^6^A modifications can be found in mRNA, rRNA and various ncRNAs, such as lncRNA, long intergenic non‐coding RNA (lincRNA), microRNA (miRNA), promoter‐associated RNA (paRNA), enhancer RNA (eRNA) and circular RNA (circRNA) (Box 1, 2, 3, 4) [112]. The sites of m^6^A marks on an RNA molecule seem to affect RNA biogenesis, processing, localization, translation and metabolism [113] (Figs 1 and 2; Box 2, 3, 4).

m^6^A is the most abundant internal modification in mammalian mRNA [114]. There are more than 7000 human transcripts that contain m^6^A [115, 116] and over 12 000 m^6^A sites are identified in the RRACH motif, with 70% and 30% frequency of ‐G‐m^6^A‐C and ‐A‐m^6^A‐C, respectively [117]. m^6^A has been widely observed in the CDS (~ 50%), 3′UTR (~ 40%) near the stop codons [116], 5′UTR (> 7%) and intronic regions (> 2%) [116]. The enriched m^6^A observed near the stop codon and in the 3′UTR suggests a definite functional role of m^6^A [116]. In addition, over 54% of mRNAs containing at least two m^6^A sites are frequently clustered in the adjacent regions of transcripts [116], suggesting a potential role of m^6^A in RNA processing.

In rRNA, two conserved m^6^A sites, m^6^A1832 in 18S rRNA and m^6^A4220 in 28S rRNA, have been identified in *X. laevis* and mammalians [118, 119]. Human rRNA modifications are introduced during ribosome biogenesis [7], where m^6^A 1832 in 18S rRNA is deposited in one of the last steps in 40S maturation. Both m^6^A modifications in rRNAs tend to localize in the functionally important regions of rRNAs, playing roles in the promotion of protein synthesis [7, 39, 120], but has no impact on overall processing or maturation of rRNA [5, 7].

m^6^A modifications have been identified in other ncRNAs. Over 700 lncRNAs with m^6^A methylation were identified [121], which is widespread in the entire body of lncRNAs and tends to be present in lncRNAs undergoing alternative splicing [122]. Over 1400 circRNAs, accounted for 54% of total circRNAs, contain m^6^A modification [123]. m^6^A is also isolated from tRNA^Val^ [19]. Despite the consensus reports show that m^6^A methylation exists on tRNA, scientists usually hard to find m^6^A abundance details on tRNA [24]. In lincRNA, the most frequent consensus motif for m^6^A deposition is GG/A(m^6^A)CH, which is slightly different from that in mRNAs [124]. Compared with unmodified lincRNAs, m^6^A‐modified lincRNAs tend to be alternatively spliced [122]. In miRNA, m^6^A modification can influence the maturation of miRNAs [125, 126]. Our previous study indicated that METTL3 can increase the splicing of precursor miR‐143‐3p to facilitate its biogenesis [127]. In addition, m^6^A could indirectly regulate the biological functions of miRNAs [112]: (a) m^6^A interferes with miRNA‐mRNA interactions by altering the RNA secondary structure of alternative polyadenylation (APA) choice in 3′UTR of targets (Box 2) [128]; (b) m^6^A could stabilize lncRNAs to act as ceRNA to regulate the activity and function of miRISC, resulting in the modulation of gene expression (Box 3) [129] and (c) miRNAs can also affect the m^6^A of targets via occupying the 3′UTR m^6^A site of mRNAs [130].

Box 3 **Functional consequences of m^6^A for lncRNA**

**Structure switch and RNA stability:** m^6^A may alter the lncRNA structure switch via interfering with the base pairing and therefore affecting its stability [131]. m^6^A methylation of A2577 and A2515 in lncRNA *MALAT1* promote its binding to HNRNPC and HNRNPG, and loss of METTL3 reduces the accessibility of *MALAT1* to HNRNPC/HNRNPG [16, 132]. A high level of m^6^A modification increases the stability of the lncRNA FAM225A [133] and METTL3 increases the stability of LINC00958 [134] and lncRNA RMBP [135] via decreasing the RNA degradation rate. In addition, m^6^A modification of *DIAPH1‐AS1* enhances its stability by relying on the IGF2BP2‐dependent pathway [136].

**Regulation of competitive endogenous RNA (ceRNA):** lncRNAs can act as miRNA sponges and mediate ceRNA to regulate the biological functions of miRNAs. On one hand, m^6^A increases the stability of lncRNAs to promote ‘sponging’ miRNAs to regulate their gene expression. For instance, LINC00958 sponges miR‐3619‐5p to increase hepatoma‐derived growth factor (HDGF) expression [134] and *MALAT1* acts as ceRNA to abolish the gene silencing function of miR‐1914‐3p [137]. On the other hand, m^6^A affects RNA‐RNA interactions via RRACU m^6^A sequence motifs interfering binding efficiency. For instance, knockdown of METTL3 suppresses the binding between linc1281 and *let‐7* miRNA, thus sequestering *let‐7* functions and regulating the differentiation of hESCs [138].

**Gene silencing and protein binding potential:** Silencing of gene transcription on the X chromosome is mediated by the lncRNA X‐inactive specific transcript (XIST). m^6^A deposition has been identified in *XIST*, which is necessary for *XIST*‐mediated transcriptional repression of X‐linked genes, such as *Gpc4* and *Atrx*, and X chromosome inactivation [139]. In addition, methylation of lncRNA *Pvt1* transcripts stabilizes the MYC protein by enhancing the *Pvt1*‐MYC interaction in epidermal progenitor cells [140].

**Subcellular localization:** m^6^A modulates the subcellular localization of lncRNA. For instance, m^6^A methylation involves in the up‐regulation of *RP11* by increasing its nuclear accumulation due to the m^6^A‐enhancing interaction of *RP11* with hnRNPA2B1 [141].

As a result, m^6^A methylation is involved in various cellular functions [142]. Increasing evidence supports that m^6^A levels are often up‐regulated in RNA molecules isolated from various cancers, and this RNA modification appears to have roles in tumorigenesis and cancer progression [143, 144]. Therefore, targeting m^6^A methylation might act as a potential approach for cancer treatment. Meanwhile, alteration of m^6^A level is being considered as a predictive biomarker for cancer diagnosis [143, 145, 146].

In this review, we first review the changes of m^6^A methylation modification and the alteration of gene expression of m^6^A writers, erasers and readers in different types of cancers. Next, we examine how m^6^A methylation is associated with tumorigenesis and cancer progression, and the possible mechanisms through which m^6^A methylation of mRNA and ncRNA targets affects tumor cell proliferation, metastasis, chemoresistance, cancer microenvironment and cancer metabolism. In addition, we discuss the potential of targeting m^6^A modifications for cancer diagnosis and therapy and highlight future challenges. In addition, we have shown the functional consequences of m^6^A modification on mRNA in Box 2.

## 2 Regulation of m^6^A writers in cancers

### 2.1 METTL3

As the major RNA m^6^A writer, the expression of METTL3 is closely associated with the genesis and development of cancers. In TCGA datasets, *METTL3* is overexpressed in a variety of cancers and shows high mutations in bladder cancer (BCA), endometrioid cancer (EOC) and colon cancer. In pancreatic adenocarcinoma (PAAD), cigarette smoke condensate induces hypomethylation of *METTL3* promoter and excessively maturates miR‐25 to promote cancer progression [147]. In CRC, butyrate, a classical intestinal microbial metabolite, can down‐regulate the expression of METTL3 to inhibit CRC development [148]. In GC, P300‐mediated H3K27 acetylation activation in the promoter region of METTL3 induces its mRNA transcription (Box 2) to promote tumor angiogenesis [149]. In lung cancer (LC), SUMOylation of METTL3 significantly represses its m^6^A MTase activity, resulting in the enhancement of tumorigenesis [150]. We previously identified the TATA‐binding protein can transcriptionally increase the expression of METTL3 in cervical cancer cells via binding to its promoter [25]. In addition, miRNAs including miR‐186 [151], miR‐4429 [152], miR‐600 [153] and let‐7g [22], are proposed to bind with *METTL3* mRNA to regulate its expression. METTL3 function in cancer is shown in Table 1.

### 2.2 METTL14

METTL14 expression is dysregulated in cancers through different mechanisms. In breast cancer (BC), METTL14 can be stabilized by AURKA by inhibiting proteasomal‐dependent degradation [154]. In AML, METTL14 expression is negatively regulated by SPI1 [28]. In CRC, KDM5C mediated demethylation of H3K4me3 in the promoter region of *METTL14* to inhibit its transcription [33]. In addition to expression dysregulation, METTL14 can be directly recruited by LNC942 to promote cancer progression of BC [29]. Interestingly, Lang et al. [155] revealed that viral‐encoded latent oncoprotein EBNA3C activated transcription of *METTL14* and directly interacted with METTL14 to enhance its stability in viral‐associated tumorigenesis. METTL14 function in cancer is shown in Table 1.

### 2.3 WTAP

WTAP, which is mainly regulated by ncRNAs in cancers, is commonly up‐regulated in many cancer types [156, 157]. In osteosarcoma, SNHG10 up‐regulates WTAP through decreasing miR‐141‐3p expression [158]. In BCA, circ0008399 binds to WTAP to promote the formation of MTC [159]. In diffuse large B‐cell lymphoma (DLBC), piRNA‐30473 up‐regulates WTAP to promote tumorigenesis [160]. Intriguingly, METTL3 regulates the homeostasis of WTAP protein via an m^6^A‐dependent manner [161]. Interestingly, m^6^A modification can stabilize *WTAPP1* RNA, which further bound its protein‐coding counterpart *WTAP* mRNA and recruited more eIF3 translation initiation complex to promote WTAP translation [162], suggesting a close crosslink between m^6^A and WTAP. WTAP function in cancer is shown in Table 1.

### 2.4 Other m^6^A writers

Less research has been done on the regulation of other m^6^A writers in cancers. For instance, Wu et al. [163] reported that ZC3H13 could be down‐regulated by miR‐362‐3p/miR‐425‐5p in hepatocellular carcinoma (HCC). Tran et al. [5] showed that METTL5 formed a heterodimeric complex with TRMT112 to gain metabolic stability. Substantial efforts are required to promote our understanding of how other m^6^A writers are modulated in cancers. Other m^6^A writers function in cancer are shown in Table 1.

Dysregulation of m^6^A writers is widely observed in different types of cancers, which has been considered to be one of the most important factors for the development of cancers. Both mRNA and ncRNA are commonly targeted by m^6^A writers in cancers, and the effects of m^6^A writers seems complex, since it can act as either promoter or suppressor to modulate the development of cancers via various mechanisms.

## 3 Regulation of m^6^A erasers in cancers

### 3.1 FTO

As the first identified RNA m^6^A demethylase, FTO is the most studied and found to be frequently dysregulated in its expression, localization, post‐translational modification and functions in various types of cancers. In CRC, hypoxia could decrease FTO expression via increasing its ubiquitin‐mediated protein degradation [47]. In EOC, the nuclear localization of FTO increases and then enhances cancer progress via the mTOR signaling pathway [164]. As to the post‐translational of FTO, p62 negatively regulates FTO stability via directly binding with FTO to facilitate the degradation of FTO protein via autophagy [165]. In AML, FTO promotes the stability of *MYC/CEBPA* transcripts and leads to the enhancement of relevant pathways [166]. Additionally, a recent study discovered that zinc finger protein 217 [167] and nicotinamide adenine dinucleotide phosphate [168] uncovered roles in FTO‐dependent adipogenic regulation. FTO function in cancer is shown in Table 1.

### 3.2 ALKBH5

Increasing research has focused on exploring the mechanisms responsible for the dysregulation of ALKBH5 in cancers: *Hypoxia*: ALKBH5 is a direct target of HIF‐1α, indicating that ALKBH5 may be involved in the regulation of cellular responses to hypoxia [169]. In addition, ALKBH5 is significantly up‐regulated under hypoxic conditions, while knockdown of HIF‐1α and/or HIF‐2α abrogates this effect in human BC cells [170]. *Histone modifications*: Wang et al. [171] found that histone demethylase KDM4C regulated ALKBH5 expression via increasing chromatin accessibility of ALKBH5 locus, by reducing H3K9me3 levels and promoting the recruitment of MYB and Pol II in AML. Qu et al. [172] identified that the highly expressed ALKBH5 was induced by HBx‐mediated H3K4me3 modification of *ALKBH5* promoter in a WDR5‐dependent manner after HBV infection. Hao et al. [173] showed that EP300‐induced H3K27 acetylation increased ALKBH5 expression in uveal melanoma (UVM). *Transcription factors*: Guo et al. [174] described that p53 interacted with the *ALKBH5* promoter, transcriptionally activating ALKBH5 and indirectly reducing m^6^A amounts in PAAD. *ncRNAs*: The lncRNA *FOXM1‐AS* enhanced ALKBH5 binding to *FOXM1* nascent mRNA in glioblastoma (GBM) cells [49]. CircRNA cIARS regulates ferroptosis in HCC cells through physically interacting with ALKBH5 [175]. ALKBH5 function in cancer is shown in Table 1.

The effect of m^6^A erasers on cancer development has been studied extensively. Similar to m^6^A writers, both m^6^A erasers play essential roles during cancer development. It's noteworthy that the expression of m^6^A erasers is sensitive to the extracellular environment such as hypoxia, hinting that m^6^A erasers might be a potential therapeutic target to increase the efficiency of novel cancer treatments such as hyperbaric oxygen therapy. In addition, expression of m^6^A erasers is commonly associated with the transcription of RNA targets and the transduction of cellular signaling, showing the global effect of m^6^A erasers in cells.

## 4 Regulation of m^6^A modification readers

### 4.1 YTH‐containing proteins

The expressions of YTH domain‐containing proteins in cancers are regulated by different mechanisms. Smoking and hypoxia conditions were demonstrated to closely correlate with the expression level of YTH proteins. YTHDC2 was significantly reduced in both LC cells and cigarette smoke‐exposed cells [176]. Hypoxia induces YTHDF2 overexpression via activation of the mTOR/AKT axis during the progression of lung squamous cell carcinoma [177]. Hypoxia can also induce a specific switch in the YTHDC1 expression pattern toward the two non‐protein coding mRNA variants [178]. HIF1α can on one hand promote the transcription activity of the *YTHDF2*, and on the other hand bind to the 5'UTR of *YTHDF2* mRNA [179]. In ocular melanoma, transcription of YTHDF2 is activated by histone acetylation [57]. It has been reported that Musashi‐1 (MSI1) up‐regulated YTHDF1 by stabilizing *YTHDF1* mRNA in GBM cells [180]. In addition, microRNAs including miR‐139‐5p [181], miR‐145 [182, 183], miR‐3436 [184], miR‐376c [185], miR‐454‐3p [186], miRNA‐495 [187] have been proposed to suppress YTH proteins by targeting their mRNAs in various cancers. YTHDF1‐3 and YTHDC1‐2 functions are shown in Table 1.

In addition, YTH proteins are also regulated by post‐translational modification. Fang et al. [188] showed that EGFR/SRC/ERK signaling phosphorylated YTHDF2 at Serine‐39 and Threonine‐381, therefore stabilizing YTHDF2 protein to promote cholesterol dysregulation and invasive growth of GBM. In contrast, Xu et al. [189] unveiled that FBW7 counteracted the tumor‐promoting effect of YTHDF2 by inducing proteasomal degradation of YTHDF2 in ovarian cancer (OV).

### 4.2 IGF2BPs

**Fig. 3:**
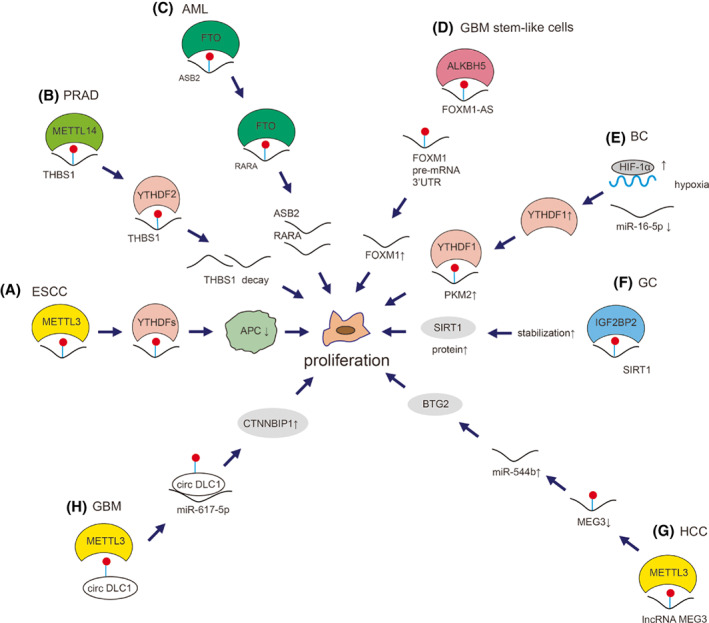
Abundances of RNA modifiers in human cancers. Comparison of expression abundance among m^6^A modifiers in different types of cancers. (A) The construction of the human body considered in different types of tumors. (B) The gene expression levels of m^6^A modifiers. The expression levels of modifiers are compared between cancer and normal tissues. Differences of which over 1.5‐fold are marked. Red plots annotated modifiers are highly expressed in tumor compared with the normal tissues, whereas green plots annotated modifiers are low expressed in tumor compared with the normal tissues. Grey plots represent that there is not enough data to identify the expression in indicated cancers. The source of the data is from the GEPIA database [200].

*IGF2BP1*: IGF2BP1 was found to be commonly and significantly up‐regulated in almost all cancer cell lines (Fig. 3) [190, 191, 192]. In HCC and GC, lncRNA HCG11 can interact with IGF2BP1 and enhance its physical interaction with c‐Myc mRNA to promote tumorigenesis [193, 194]. In human intrahepatic cholangiocarcinoma, miR‐885‐5p promotes the down‐regulation of IGF2BP1 to inhibit cell proliferation and metastasis [195]. *IGF2BP2*: HMGAs are crucial for the expression of IGF2BP2. HMGA1 suppressed the expression of IGF2BP2, which in turn bound and stabilized *HMGA1* mRNA to promote cell proliferation [196]. HMGA2 can also promote IGF2BP2 transcription by binding to the AT‐rich region of the *IGF2BP2* gene in cooperation with NF‐κB [197]. In addition, Lai et al. [198] unveiled that IGF2BP2 activity could be mediated by mTOR, a major effector downstream of PI3K/Akt signaling. *IGF2BP3*: Similar to IGF2BP1, a major mechanism of IGF2BP3 regulation is based on its complex interaction with the ncRNA machinery. For example, hsa_circ_0003258 is physically bound to IGF2BP3 in the cytoplasm to activate ERK signaling pathway in prostate cancer (PRAD) [76]. circIGHG directly binds with miR‐142‐5p and consequently elevates IGF2BP3 activity in oral squamous cell carcinoma [199]. IGF2BP1‐3 function are shown in Table 1.

### 4.3 hnRNPCs

hnRNPCs including hnRNPA2/B1, HNRNPC, HNRNPE and HNRNPH are found to be prevalently and significantly up‐regulated expression in a variety of tumors associated with cancer cells metastasis [77, 78, 79, 80]. *hnRNPA2/B1 and HNRNPC*: both hnRNPA2/B1 and HNRNPC are up‐regulated in tumors [201]. However, their up‐regulated mechanisms remain to be elucidated [80]; hNPNPCs could directly bind with oncogenes to control tumorigenesis, including regulating RNA splicing, RNA exportation, RNA expression, RNA stability and translation (Box 2) [78, 202, 203]. *HNRNPE*: For instance, Breege et al. [79] demonstrated that E3 ubiquitin ligase ARIH1 could regulate hnRNP‐E1 to promote BC cells invasion. *HNRNPH*: HNRNPH could interact with a broad of target to act as splicing factor in tumor progression. The functions of hnRNPRs are shown in Table 1.

m^6^A readers are the executors of m^6^A marks, leading to various regulatory effects on targets and, therefore, affecting the cellular events. It is worth to notice that the relationship between m^6^A readers and RNAs are not straightforward. On the one hand, m^6^A readers can modulate the expression and/or biological functions of RNAs such as via RNA‐RNA interaction. On the other hand, the activity or expression of m^6^A readers can be regulated by RNAs. Although increasing studies show the importance of m^6^A readers in the development of cancers, the detailed mechanisms of m^6^A readers and the cooperations among different m^6^A readers need to be further explored.

## 5 The m^6^A modification in cancer cell proliferation

### 5.1 Regulation via m^6^A on mRNAs

**Fig. 4:**
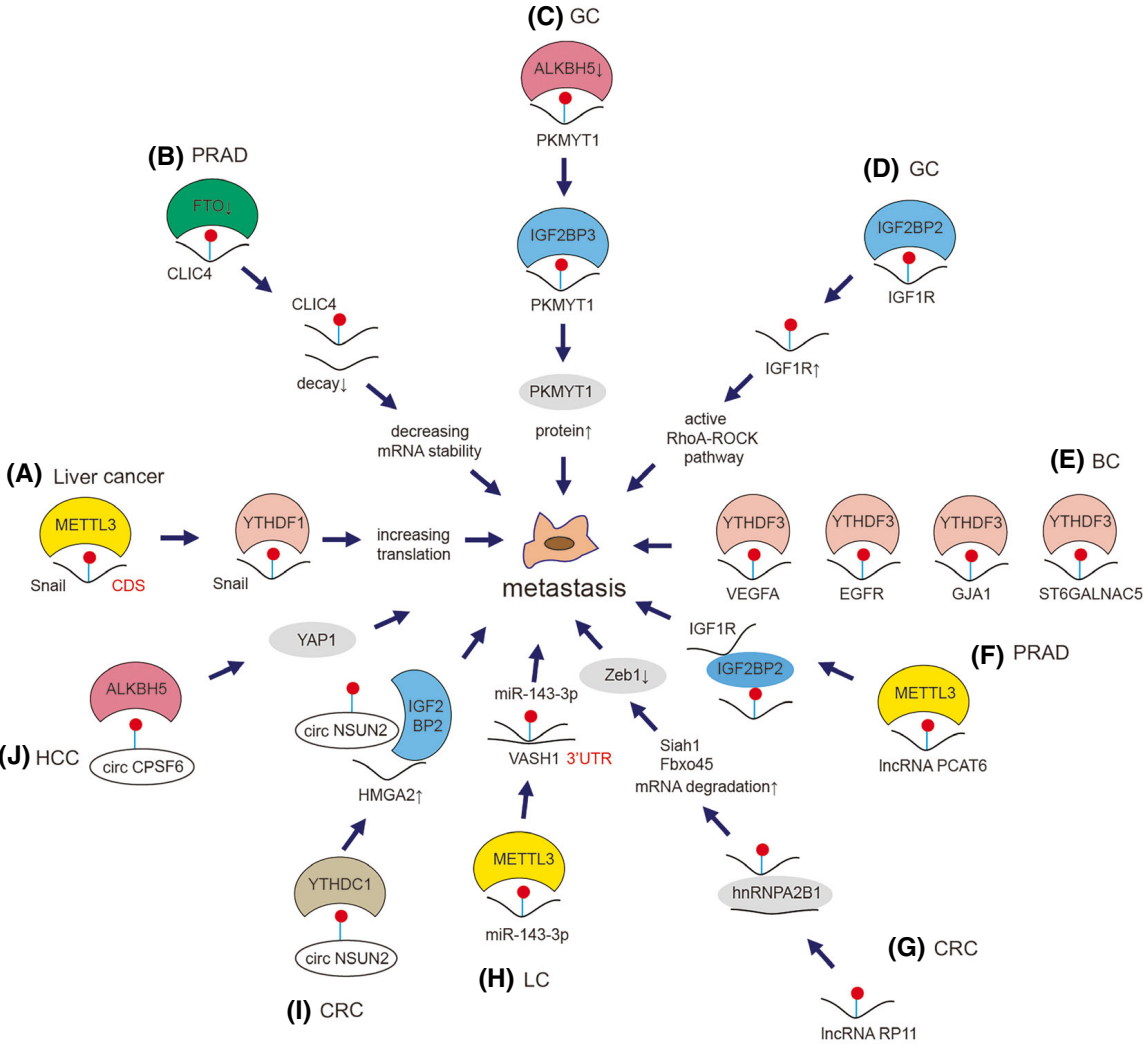
Mechanism of m^6^A on cancer proliferation. m^6^A modulates the proliferation via various mechanisms in cancers. (A) METTL3‐mediated deposition of m^6^A decreases APC expression with YTHDFs binding in ESCC cells [204]. (B) METTL14‐mediated m^6^A modification on *THBS1* mRNA promotes YTHDF2‐mediated *THBS1* decay in PRAD cells [205]. (C) FTO‐mediated m^6^A modification on both *ASB2* and *RARA* mRNA suppresses their expression in AML [44]. (D) ALKBH5 removes m^6^A on lncRNA FOXM1‐AS facilitating the interaction between FOXM1 3′UTR and ALKBH5 to promote the expression of FOXM1 in GBM stem‐like cells [49]. (E) tumor hypoxia induces HIF‐1α and decreases miR‐16‐5p level, resulting in the up‐regulation of YTHDF1 to promote the YTHDF1‐mediated PKM2 expression in BC cells [206]. (F) IGF2BP2 recognizes m^6^A on *SIRT1* mRNA and stabilizes SIRT1 in GC cells [207]. (G) METTL3 deposits m^6^A on lncRNA MEG3, down‐regulating MEG3 levels and up‐regulating miR‐544 and, therefore, regulates BTG2 expression to represses proliferation of HCC cells [208]. (H) METTL3‐mediated m^6^A upregulates circDLC1 expression and the interaction between circDLC1 and miR‐671‐5p and, therefore, promotes CTNNBIP1 expression in GBM cells [209].

METTL3 can promote the cell proliferation of esophageal squamous cell carcinoma (ESCC) by decreasing APC expression mediated by *APC* mRNA m^6^A‐dependent YTHDFs binding (Fig. 4A) [204]. METTL14 can promote PRAD cell proliferation by inhibiting THBS1 via an m^6^A‐YTHDF2‐dependent mechanism (Fig. 4B) [205]. FTO targets and suppresses the expression of *ASB2* and *RARA* mRNA to promote cell proliferation and viability in AML (Fig. 4C) [44]. ALKBH5 demethylates the nascent transcripts of *FOXM1* mRNA to enhance its expression, leading to the promotion of proliferation and tumorigenesis of GBM stem‐like cells (Fig. 4D) [49]. YTHDF1 mediates cell growth and metastasis of BC through regulating *PKM2* mRNA to affect glycolysis (Fig. 4E) [206]. IGF2BP2 regulates the proliferation/migration of GC by recognizing the m^6^A modification sites of *SIRT1* mRNA (Fig. 4F) [207].

### 5.2 Regulation via m^6^A on ncRNAs

Wu et al. [208] showed that m^6^A‐induced lncRNA MEG3 suppressed the proliferation, migration and invasion of HCC cells through miR‐544b/BTG2 signaling (Fig. 4G). Wu et al. [209] determined that METTL3‐mediated m^6^A modification up‐regulated circDLC1 expression and promoted *CTNNBIP1* transcription by sponging miR‐671‐5p, thus repressing the malignant proliferation of GBM (Fig. 4H).

The relationship between m^6^A modification and cancer cell proliferation has been drawing attention in recent years. The regulation and/or role of m^6^A in cell proliferation appears to be cancer type‐dependent. Furthermore, the regulatory effects of m^6^A on cell proliferation can be achieved through different mRNAs or ncRNAs, which could be positive or negative, mainly dependent on the m^6^A targets. Nevertheless, YTHDFs play more essential roles in the regulation of cell proliferation than other m^6^A readers.

## 6 The m^6^A modification in metastasis

### 6.1 Regulation via m^6^A on mRNAs

**Fig. 5:**
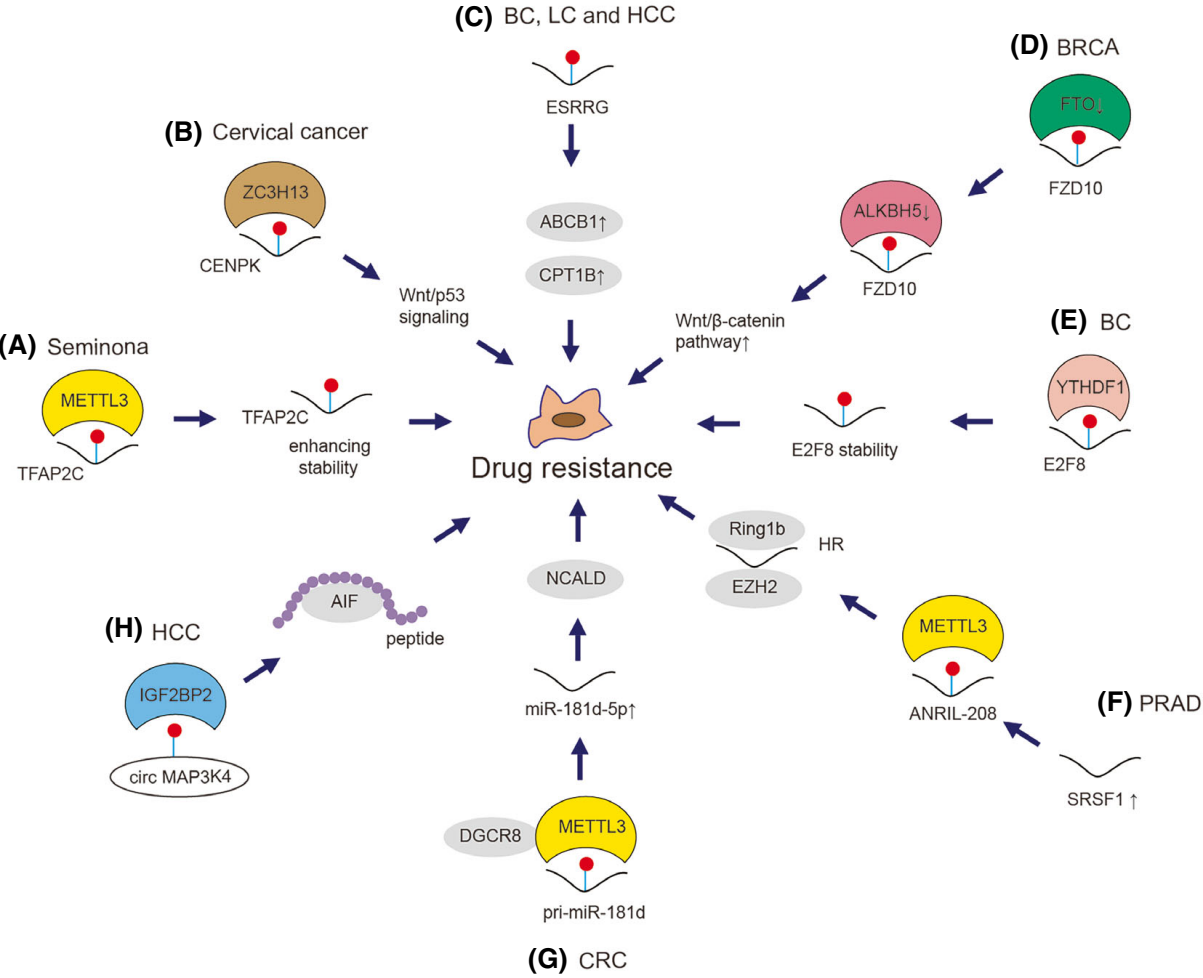
Mechanism of m^6^A on cancer metastasis. m^6^A modulates metastasis via various mechanisms in cancers. (A) METTL3 deposits m^6^A on CDS of *Snail* mRNA and then targeted by YTHDF1 to increase its translation to mediate metastasis in liver cancer [101]. (B) FTO‐mediated demethylation of m^6^A on *CLIC4* mRNA decreases its stability, resulting in the repression of metastasis in PRAD cells [210]. (C) IGF2BP3 helps stabilize the mRNA stability of *PKMYT1* via an ALKBH5‐dependent manner to regulate metastasis in GC cells [211]. (D) IGF2BP2 recognize m^6^A on *IGF1R* mRNA and increase its expression to activate RhoA‐ROCK pathway and therefore promote metastasis in GC cells [212]. (E) YTHDF3 promotes metastasis by inducing the translation of *ST6GALNAC5*, *GJA1*, *EGFR* and *VEGFA* mRNAs in BC cells [60]. (F) METTL3 promotes metastasis by methylating lncRNA PCAT6, which recognized by IGF2BP2 to stabilize IGF2BP2/IGF1R interaction in PRAD cells [73]. (G) m^6^A‐modificed lncRNA RP11 forms complex with hnRNPA2B1, accelerating the mRNA degradation of *Siah1* and *Fbxo45* to mediate metastasis by targeting of Zeb1 in CRC cells [141]. (H) m^6^A‐modificed miR‐143‐3p binds to the 3′UTR of *VASH1* to promote metastasis in LC cells [127]. (I) YTHDC1 recognizes m^6^A‐modified circNSUN2 to enhance the circNSUN2/ *HMGA2*/IGF2BP2 interaction to promote metastasis in CRC cells [213]. (J) ALKBH5‐mediated demethylation of circCPSF6 promotes metastasis by activating YAP1 in HCC cells [214].

We previously highlighted that m^6^A was critical in the progress of epithelial–mesenchymal transition (EMT) since *Snail* could be modified by m^6^A in the CDS region and METTL3/YTHDF1 could mediate the expression and translation of *Snail* mRNA to regulate cancer cells growth and metastasis (Fig. 5A) [101]. Zou et al. [210] demonstrated that FTO suppressed PRAD cell proliferation and metastasis by reducing the degradation of *CLIC4* mRNA in an m^6^A‐dependent manner (Fig. 5B). Hu et al. [211] found that ALKBH5 suppressed the invasion of GC via *PKMYT1* m^6^A modification (Fig. 5C). IGF2BP2 increased the expression of *IGF1R* by identifying m^6^A modification sites in *IGF1R* mRNA, thus activating the RhoA‐ROCK pathway to promote GC metastasis (Fig. 5D) [212]. YTHDF3 induced the translation of m^6^A‐enriched gene transcripts such as *ST6GALNAC5* and *GJA1* to promote metastasis of BC in the brain (Fig. 5E) [60].

### 6.2 Regulation via m^6^A on ncRNAs

Lang et al. [73] showed that m^6^A‐modified lncRNA PCAT6 stabilized IGF2BP2/IGF1R to promote PRAD bone metastasis and tumor growth (Fig. 5F). We previously identified that m^6^A‐induced lncRNA RP11 triggered the dissemination of CRC cells via up‐regulation of Zeb1 (Fig. 5G) [141]. We found that m^6^A‐induced miR‐143‐3p promoted the brain metastasis of LC via regulation of VASH1 (Fig. 5H) [127]. Chen et al. [213] elucidated that m^6^A modification of circNSUN2 modulated the cytoplasmic export and stabilized *HMGA2* to promote liver metastasis of CRC (Fig. 5I, Box 4). Furthermore, m^6^A‐modified circCPSF6 triggered the metastasis of HCC cells via activation of YAP1 (Fig. 5J) [214] (Table 2).

Box 4 **Functional consequences of m^6^A for circRNA**

**Biogenesis:** circRNA biogenesis requires the back splicing, which occurs at the m^6^A‐enriched sites for a subset of circRNAs. These m^6^A‐enriched sites are usually located around the start and stop codons in linear mRNAs [222]. A recent study also revealed that METTL3 and YTHDC1 could regulate the biogenesis of circ‐ZNF609 via regulating circ‐ZNF609 level [223].

**Degradation and stability:** Deposition of m^6^A on circRNA have dual effect on the regulation of circRNA stability: promotes degradation and enhances stability. m^6^A in circRNA can be recognized by YTHDF2, which recruits the RNase R/MRP complex to cleave circRNA, and therefore promotes the degradation of circRNA [224]. Conversely, m^6^A stabilizes the expression of circCUX1 [225] and circRNA‐SORE [226]. It is likely that the m^6^A‐regulated circRNA stability is dependent on the recognition of different m^6^A readers or the deposition location of m^6^A in circRNA.

**Initiation of extensive translation:** Most of the circRNAs are ncRNAs, which fail to recruit translation initiation complexes due to a lack of 5′UTR and m^7^G cap. However, some circRNAs can be m^6^A modified and recognized by YTHDF3, which therefore recruit the pre‐initiation complex to circRNAs. This m^6^A‐mediated extensive translation of circRNAs is cap‐independent. Nowadays, over a hundred peptides produced by circRNAs have been identified in germ cells [227]. YTHDF3 and eIF4G2 are physically associated with endogenous circ‐ZNF609 and are essential for its translation driven by m^6^A [223].

**Cytoplasmic export:** m^6^A‐modified circRNA, circNSUN2, could be recognized by YTHDC1 and facilitate its export to cytoplasm [213]. Cytoplasmic circNSUN2 can form an RNA‐protein ternary complex with IGF2BP2 and high mobility group protein 2 (HMGA2), which stabilizes *HMGA* mRNA and promotes metastasis of CRC [213].

**Regulation of biological functions:** circRNAs often act as miRNA ‘sponges’. m^6^A on circRNA influences the binding between circRNA and miRNA, thereby affecting the miRNA‐silencing functions on target mRNAs [123] or sequestering target miRNAs in the cytoplasm [228]. m^6^A depositions on circRNA can be used as markers to identify ‘self’ and ‘foreign’ circRNA during viral defense [229]. For instance, circE7 from the HPV virus can be modified by m^6^A and labeled as ‘self’ circRNA, which facilitates the virus's escape from the host antiviral immune response [229].

**Table 2:** Non‐coding RNA influenced by m^6^A and its function in cancers.

Type	Name	Effect	Mechanisms
circRNA	circ0008399	Promotes cell cisplatin resistance (BCA)	Up‐regulation of TNFAIP3 [159]
	circ_104075	Stimulates YAP‐dependent tumorigenesis (HCC)	Up‐regulation of YAP by absorbing miR‐582‐3p [215]
	circDLC1	Inhibits MMP1‐mediated cancer progression (LC)	Interaction with HuR and down‐regulation of MMP1 [41]
miRNA	miR‐25‐3p	Promotes cancer progression (PRAD)	Activation of AKT‐p70S6K signaling [147]
	miR‐96	Promotes cancer occurrence and progression (CRC)	Regulation of AMPKα2‐FTO‐m^6^A/MYC axis [216]
	miR‐143‐3p	Promotes lung cancer brain metastasis (LC)	Inhibition of VASH1 [127]
	miR‐320b	Inhibits cancer angiogenesis and tumor growth (LC)	Inhibition of HNF4G, IGF2BP2 and TK1 [217]
	miR‐135	Inhibits cell epithelial–mesenchymal transition (BC)	Regulation of miR‐135/ZNF217/METTL3/NANOG axis [218]
lncRNA	FAM225A	Promotes tumorigenesis and metastasis (NPC)	Adsorption of miR‐590‐3p and miR‐1275 and up‐regulation of ITGB3 [133]
	LCAT3	Promotes tumorigenesis (LC)	Activation of c‐MYC [219]
	LINC00278	Inhibits cell apoptosis (ESCC)	Down‐regulation of YY1BM [220]
	GAS5	Inhibits cancer progression (CRC)	Phosphorylation and degradation of YAP [221]
rRNA	28S	Inhibits cell proliferation (HCC)	Reduction of global translation [7]
	18S	Promotes cell proliferation (BC)	Promotion of translation initiation [39]

Metastasis is a major cause of cancer mortality, but its molecular mechanisms are severely understudied. Increasing research reveals the link between m^6^A and metastasis, showing that m^6^A may help modulate metastasis in cancer progression via different mechanisms. Among them, promotion of translation seems to be the major effect of m^6^A on the metastasis process, since YTHDF1/3 and IGF2BP2/3 are commonly involved. Despite mRNA, ncRNA including circRNA, lncRNA and miRNA are contributed to the regulation of metastasis, most of them are related to the up‐regulation of targets that promote metastasis.

## 7 The m^6^A modification in chemoresistance

### 7.1 Regulation via m^6^A on mRNAs

**Fig. 6:**
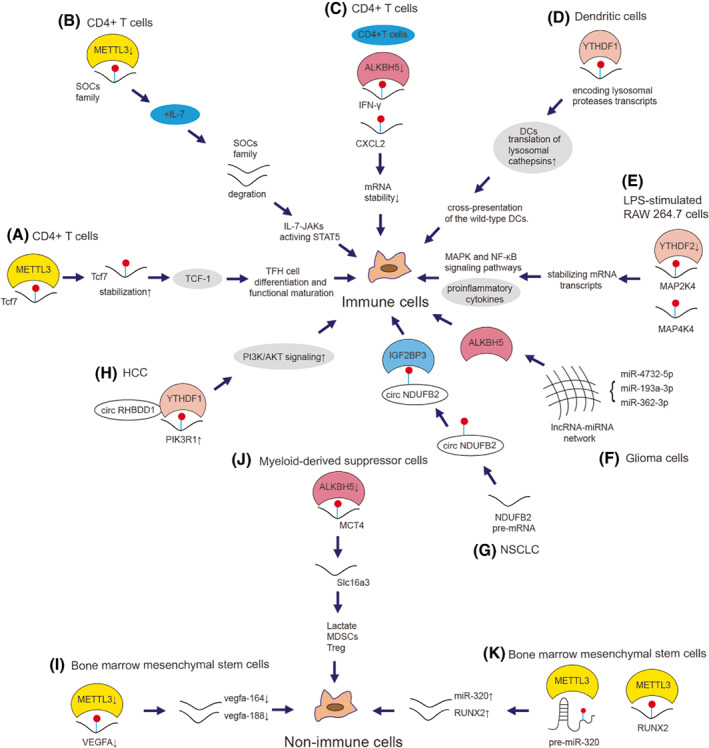
Mechanism of m^6^A on cancer cell drug resistance. m^6^A modulates cancer cell drug resistance via various mechanisms in cancers. (A) METTL3 methylates *TFAP2C*, which enhances the stability of *TFAP2C* to increase chemoresistance in seminoma [230]. (B) ZC3H13 targets m^6^A on *CENPK* to activates Wnt/p53 signaling and therefore enhances chemoresistance in cervical cancer [231]. (C) m^6^A‐modified *ESRRG* mRNA upregulates protein expression of both ABCB1 and CPT1B to enhance chemoresistance in BC, LC and HCC cells [232]. (D) Down‐regulation of either ALKBH5 or FTO promotes m^6^A deposition on *FZD10* mRNA, which activates Wnt/β‐catenin pathway to enhance chemoresistance in BRCA cells [233]. (E) YTHDF1 recognize m^6^A on *E2F8*, modulating *E2F8* mRNA stability to enhance chemoresistance in BC cells [234]. (F) Upregulation of SRSF3 promotes ANRIL splicing and m^6^A modification of ANRIL in PRAD cells. ANRIL‐208 (one of the ANRIL spliceosomes) can enhance DNA homologous recombination repair (HR) capacity by forming a complex with Ring1b and EZH2, which enhances chemoresistance [235]. (G) METTL3‐dependent m^6^A modification of pri‐miR‐181d promotes miR‐181b‐5p process by DiGeorge Syndrome Critical Region 8 (DGCR8). miR‐181b‐5p directly targets neurocalcin δ (NCALD) to enhance chemoresistance in CRC cells [236]. (H) IGF2BP1 recognized the circMAP3K4 m^6^A modification and promotes its translation into a novel peptide, which interacts with AIF to prevent cisplatin‐induced apoptosis in HCC [237].

Wei et al. [230] showed that METTL3 enhanced the stability of *TFAP2C* mRNA by m^6^A modification in seminoma to potentiate resistance to cisplatin (Fig. 6A). Lin et al. [231] found that ZC3H13‐mediated m^6^A modification of *CENPK* mRNA promoted cervical cancer stemness and chemoresistance (Fig. 6B). We previously found that m^6^A can trigger the splicing of precursor *ESRRG* mRNA to confer chemoresistance of cancer cells through up‐regulation of ABCB1 and CPT1B (Fig. 6C) [232]. Fukumoto et al. [233] elucidated that down‐regulation of ALKBH5 and FTO increased m^6^A modified of *FZD10* mRNA contributed to PARP inhibitors resistance in BRCA‐deficient epithelial ovarian cancers cells via up‐regulation of Wnt/β‐catenin pathway (Fig. 6D). YTHDF1 modulates *E2F8* mRNA stability to promote BC cell growth, DNA damage repair and chemoresistance (Fig. 6E) [234].

### 7.2 Regulation via m^6^A on ncRNAs

Wang et al. [235] found that the lncRNA ANRIL splicing is m^6^A modification‐related, which is mediated by SRSF3 and leads to the gemcitabine‐resistance of PRAD (Fig. 6F). Pan et al. [236] reported that METTL3‐dependent m^6^A methylation increased miR‐181d‐5p expression, then inhibited the 5‐Fluorouracil sensitivity of CRC cells by targeting neurocalcin δ (Fig. 6G). Duan et al. [237] demonstrated that m^6^A‐modified circMAP3K4 could encode a novel peptide to prevent apoptosis in HCC (Fig. 6H; Table 2)

Cancer cells gradually develop resistance to progressive chemotherapy, resulting in treatment failure that has become a serious clinical problem in cancer therapy. m^6^A modification has been reported to be involved in cancer cells developing drug resistance by regulating target either transcript level or translation. Unlike the dual effect of m^6^A modification on cell proliferation, m^6^A commonly promote the chemoresistance of cancer cells, since up‐regulation of METTL3 and down‐regulation of FTO/ALKBH5 are frequently observed in drug resistance cancer cells, hinting that targeting m^6^A might be a feasible direction for drug resistant cancer therapy.

## 8 The m^6^A modification and the tumor microenvironment

### 8.1 Regulation via m^6^A on mRNAs in immune cells

**Fig. 7:**
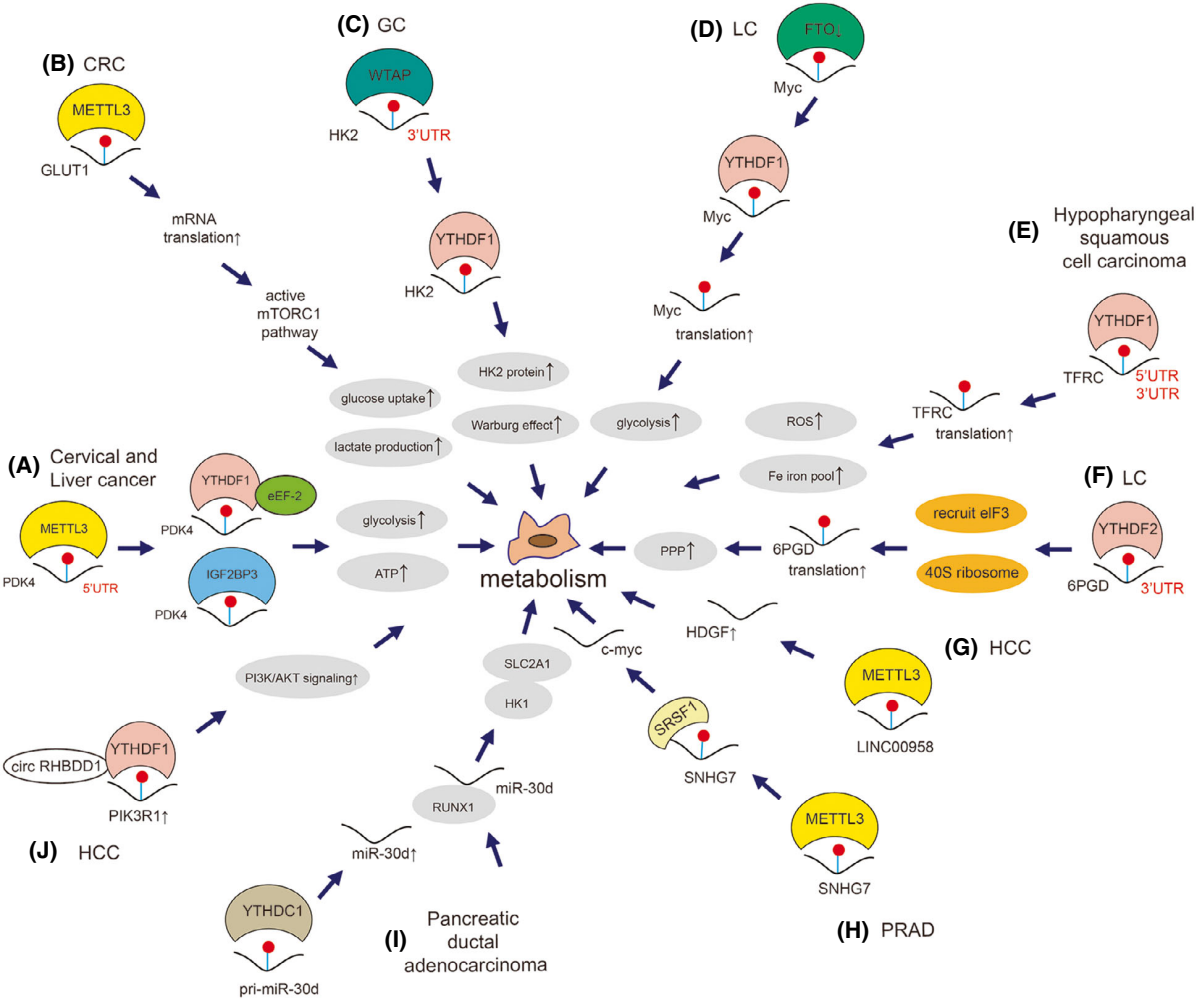
Mechanism of m^6^A on immune cells and non‐immune cells. m^6^A is involved in immunity via various mechanisms in cancers. (A) METTL3 methylates and stabilizes *Tcf7* mRNA to enhance TCF‐1 level, which promotes TFH cell differentiation/function maturation to inhibit the antibody response of B cells in CD4^+^ T cells [238]. (B) Deficiency of METTL3 stabilizes *SOCS* family expression, which inhibits IL‐7‐mediated STAT5 activation and T‐cell homeostatic proliferation and differentiation in CD4^+^ T cells [239]. (C) ALKBH5 deficiency decreases *IFN‐γ* and *CXCL2* expression to alleviate experimental autoimmune encephalomyelitis in CD4^+^ T cells [240]. (D) YTHDF1 downregulates the anti‐tumor immune responses by enhancing the translation of lysosomal proteases related mRNA in Dendritic cells [241]. (E) YTHDF2 knockdown increases the expression and stability of *MAP2K4* and *MAP4K4* mRNAs and, therefore, activates MAPK and NF‐κB signaling pathways to promote the expression of proinflammatory cytokines and aggravate the inflammatory response in LPS‐stimulated RAW 264.7 cells [242]. (F) lncRNA‐miRNA network such as miR‐4732, miR‐193a‐3p and miR‐362‐3p regulates ALKBH5 expression to recruit the M2 macrophage to glioma cells [245]. (G) m^6^A‐modified circNDUFB2 inhibits the progression of NSCLC via destabilizing IGF2BPs to activate anti‐tumor immunity [246]. (H) circRHBDD1 recruits YTHDF1 to the m^6^A‐modifed *PIK3R1* mRNA and accelerates its translation to restrict anti‐PD‐1 therapy via activation of PI3K/AKT signaling in HCC [247]. (I) METTL3 knockdown decrease *VEGFA* expression especially two transcripts vegfa‐164 and vegfa‐188 to inhibit osteogenic differentiation in bone marrow mesenchymal stem cells [243]. (J) ALKBH5 targets MCT4 to modulate Mct4/Slc16a3 expression to regulate the composition of tumor‐infiltrating Treg, level of lactate and myeloid‐derived suppressor cells [244]. (K) METTL3 targets both *RUNX2* and precursor‐miR‐320 to increase their expression, which controls the osteogenic potential of bone marrow–derived mesenchymal stem cells [248].

METTL3 in CD4^+^ T cells stabilizes *Tcf7* mRNA to prevent their differentiation and functional maturation, further inhibiting the antibody response of B cells (Fig. 7A) [238]. METTL3 can also inhibit T‐cell homeostatic proliferation and differentiation by stabilization of the mRNAs of *SOCS* pLfamily, which are the STAT signaling inhibitory proteins (Fig. 7B) [239]. During the induced neuroinflammation, ALKBH5 deficiency in CD4^+^ T cells decreases the mRNA stability of *IFN‐γ* and *CXCL2*, thereby alleviating experimental autoimmune encephalomyelitis (Fig. 7C) [240]. YTHDF1 enhances the translation of mRNAs that encode lysosomal proteases, which can degrade antigens in lysosomes to down‐regulate the anti‐tumor immune responses of dendritic cells (Fig. 7D) [241]. YTHDF2 knockdown increases *MAP2K4* and *MAP4K4* expression levels via stabilizing mRNA transcripts, which activates MAPK and NF‐κB signaling pathways to promote the expression of proinflammatory cytokines (Fig. 7E) [242]. On the other hand, when it comes to non‐immune cells, METTL3 knockdown inhibits osteogenic differentiation and alternative splicing of *VEGFA* in bone marrow mesenchymal stem cells (Fig. 7I) [243]. ALKBH5 can modulate Mct4/Slc16a3 expression and lactate content of the tumor microenvironment to regulate the composition of tumor‐infiltrating Treg and myeloid‐derived suppressor cells (Fig. 7J) [244].

### 8.2 Regulation via m^6^A on ncRNAs in immune cells

Expression of ALKBH5 can be regulated by lncRNA‐miRNA network containing miR‐4732‐5p, miR‐193a‐3p and miR‐362‐3p, which can recruit the M2 macrophage to glioma cells (Fig. 7F) [245]. circNDUFB2 inhibits the progression of NSCLC via destabilizing IGF2BPs to activate anti‐tumor immunity (Fig. 7G, Box 4) [246]. Cai et al. [247] found that CircRHBDD1 restricted PD‐L1 immunotherapy efficacy via m^6^A modification in HCC (Fig. 7H). In terms of non‐immune cell m^6^A regulation such as bone marrow mesenchymal stem cells, Yan et al. [248] demonstrated that METTL3 controlled the osteogenic potential of bone marrow‐derived mesenchymal stem cells by m^6^A methylation of precursor‐miR‐320/RUNX2 (Fig. 7K). The underlying effects of regulation of m^6^A on ncRNAs in the TME should be further explored. (Table 2)

The tumor microenvironment consists mainly of an immune microenvironment dominated by immune cells and a non‐immune microenvironment dominated by fibroblasts, formed by the combined action of malignant tumor cells and non‐transformed cells [249]. Roles of the m^6^A modification in both immune cells and non‐immune cells in the cancer microenvironment have been studied. However, the regulatory effects of m^6^A on cancer microenvironment is controversial, especially for the roles of METTL3 and ALKBH5 in immune cells and non‐immune cells. Since cancer microenvironment is special and complex, the multiple effect/roles of m^6^A modification requires further exploration.

## 9 The m^6^A modification and cancer metabolism

### 9.1 Regulation via m^6^A on mRNAs

**Fig. 8:**
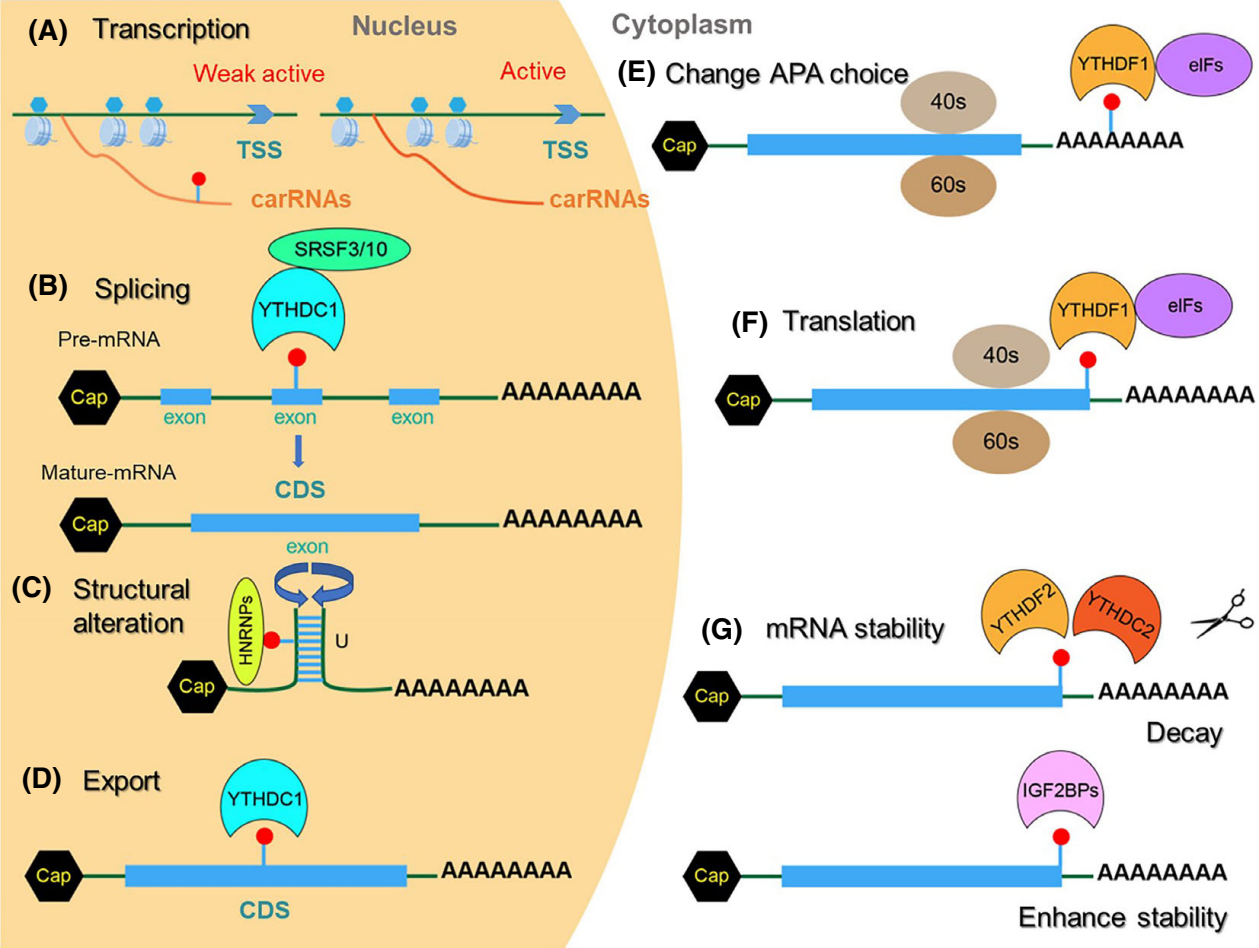
Mechanism of m^6^A on cancer cell metabolism. m^6^A modulates metabolism via various mechanisms in cancers. (A) METTL3‐modified m^6^A on 5′UTR of *PDK4* is recognized by YTHDF1/eEF‐2 and IGF2BP3 to promote the glycolysis and ATP generation in both cervical and liver cancer cells [25]. (B) METTL3 targets *GLUT1* to increase its mRNA, which actives mTORC1 pathway to promote glucose uptake and lactate production in CRC cells [250]. (C) WTAP targets 3′UTR of *HK2* to enhance its stability, which is recognized by YTHDF1 to promote glycolytic capacity in GC cells [251]. (D) FTO downregulation promotes the YTHDF1‐medicated translation of *MYC*, which increases glycolysis in LC cells [252]. (E) YTHDF1 recognizes both 5′UTR an 3′ UTR m^6^A of *TFRC*, promoting its translation to enhance iron metabolism in hypopharyngeal squamous cell carcinoma [53]. (F) YTHDF2 binds to *6PGD* mRNA to facilitate its translation, which enhances the activity of the PPP flux in LC cells [253]. (G) METTL3 increases HDGF‐involved lipogenesis by upregulating lncRNA LINC00958 in HCC cells [134]. (H) METTL3 targets lncRNA SNHG7 to increase its mRNA level. SNHG7 regulated c‐Myc via interacting with SRSF1 to promote glycolysis via SRSF1/c‐Myc axis in PRAD cells [254]. (I) YTHDC1 promotes the maturation of miR‐30d to increase its expression, which further regulates the expression of RUNX1, SLC2A1 and HK1 and therefore attenuates the Warburg effect in pancreatic ductal adenocarcinoma cells [62]. (J) circRHBDD1 recruits YTHDF1 to the m^6^A‐modifed *PIK3R1* mRNA and accelerates its translation to augment aerobic glycolysis via activation of PI3K/AKT signaling in HCC [247].

We previously showed that METTL3‐modified 5′UTR of *PDK4* mRNA could positively regulate the glycolysis and ATP generation in cervical and liver cancer cells (Fig. 8A) [25]. METTL3 enhanced *GLUT1* mRNA translation in an m^6^A‐dependent manner to promote glucose uptake and lactate production in CRC (Fig. 8B) [250]. WTAP enhances the stability of *HK2* mRNA through binding with its 3′UTR m^6^A site, leading to the promotion of GC cell proliferation and glycolytic capacity (Warburg effect) (Fig. 8C) [251]. Down‐regulated FTO in LC cells promoted the translation of *MYC* mRNA and increased glycolysis and cancer progression (Fig. 8D) [252]. YTHDF1 could regulate the translation of *TFRC* mRNA by binding its 3′ and 5'UTR to enhance iron metabolism in hypopharyngeal squamous cell carcinoma (Fig. 8E) [53]. YTHDF2 could directly bind to the 3′UTR of *6PGD* mRNA to promote its translation, therefore enhancing the activity of the pentose phosphate pathway (PPP) flux in LC cells (Fig. 8F) [253].

### 9.2 Regulation via m^6^A on ncRNAs

METTL3 mediated the up‐regulation of lncRNA LINC00958 through stabilizing its transcript and increasing lipogenesis, which could act as a nanotherapeutic target in HCC (Fig. 8G, Box 3) [134]. Liu et al. [254] found that METTL3‐stabilized lncRNA SNHG7 accelerated glycolysis in PRAD via the SRSF1/c‐Myc axis (Fig. 8H). YTHDC1 promoted the maturation of miR‐30d to suppress aerobic glycolysis by binding RUNX1, regulating SLC2A1 and HK1 expression, thus attenuating the Warburg effect to inhibit tumor progression in pancreatic ductal adenocarcinoma (Fig. 8I) [62]. circRHBDD1 was revealed to augment aerobic glycolysis in HCC (Fig. 8K) [247].

Recently, the relationship between m^6^A modification and cancer metabolism has received attention. Increasing reports suggest that m^6^A modification is extensively involved in the metabolic regulation of tumors. Compared with m^6^A erasers, m^6^A writers, especially METTL3, plays more critical roles in the regulation of cancer metabolism. In addition, m^6^A‐promoted translation is important for the glycolysis of cancer cells. On one hand, it hints that glycolysis of cancer cells could be regulated by multiple pathways. On the other hand, targeting the m^6^A‐modifed translation may be a potential approach to inhibit cancer metabolism, and therefore achieving efficient treatment of cancers.

## 10 m^6^A modifications as diagnostic and therapeutic targets

m^6^A is commonly up‐regulated in several cancers and promotes tumorigenesis. Targeting m^6^A is emerging as a new trend for cancer diagnosis and therapy due to the specific induction of m^6^A by cancer tissues and the critical effects of m^6^A on cancer development. Here, we summarized the development of potential cancer diagnosis and therapy methods by targeting m^6^A.

### 10.1 m^6^A as biomarkers for cancer diagnosis

#### 10.1.1 Total m^6^A

m^6^A level in blood/serum could be measured as simply noninvasive biomarkers for cancers. For instance, Pei et al. [145] found that leukocyte m^6^A was significantly elevated in non–small cell lung cancer (NSCLC) patients, which was suitable for NSCLC monitoring and diagnosis. In GC patients, we found that the level of m^6^A in peripheral blood RNA increased significantly. The sensitivity of for m^6^A, estimated by the value of area under the curve (AUC), in the GC group was 0.929 (95% confidence interval (CI), 0.88–0.96), which was markedly greater than the AUCs for carcinoembryonic antigen (CEA; 0.694) and carbohydrate antigen 199 (CA199; 0.603). It indicated that the level of m^6^A in peripheral blood RNA was a promising noninvasive diagnostic biomarker for GC [143]. Similarly, the m^6^A levels in peripheral blood leukocytes could be a noninvasive biomarker for both NSCLC [145] and CRC [146].

#### 10.1.2 m^6^A‐related RNAs

Over 138 m^6^A‐related transcripts were identified to be potential prognostic biomarkers so far, such as *NMPM1* in lung adenocarcinoma [255], *SNRPC* in HCC [256], *GLUT1* in esophageal cancer [257], *BATF2* in GC [258], *PGM1* and *ENO1* in BCA [259], *NUF2/CDCA3/KIF14* in clear cell renal cell carcinoma [260]. m^6^A‐associated miRNAs are also used for developing new cancer biomarkers. Zhang et al. [261] demonstrated that the m^6^A‐miRNA signatures showed superior sensitivities in each cancer type and presented a satisfactory AUC in identifying LC, GC and HCC; m^6^A‐related lncRNAs have also been identified as cancer biomarkers. For instance, 12 m^6^A‐related lncRNAs in lung adenocarcinoma (LUAD) [262] and 6 m^6^A‐related lncRNAs in BC [263] were identified as promising predictive biomarkers. In addition, specific lncRNAs including circ3823 and circ1662 in CRC [264, 265], LINC00022 in ESCC [266], circRNA_104075 in HCC [215] and MIR497HG/FENDRR/RP1‐199J3 in LUAD [267] were suggested for diagnosis.

#### 10.1.3 m^6^A regulators

The abundance of m^6^A‐related writers, erasers and readers could be candidates for tumor diagnosis. For instance, METTL3 is suggested to be a prognostic and immune‐related biomarker in BCA [268], while METTL14 is correlated with prognosis in rectal cancer patients and immune infiltration level [269]. Demethylase ALKBH5 is up‐regulated in several solid tumors and can be a biomarker for some malignant tumor prognosis, such as NSCLC and CRC [245]. Similarly, FTO [270], WTAP [271], KIAA1429 [272], RBM15 [273], ZC3H13 [274], METTL5 [275], METTL16 [274], ZCCHC4 [276], HNRNPC [276] YTHDF1 [277], YTHDF2 [278, 279], YTHDF3 [280, 281], YTHDC1 [282], YTHDC2 [56], IGF2BP1 [283, 284], IGF2BP2 [285, 286], IGF2BP3 [287] have been reported to be potential biomarkers for prognosis in different cancers.

These studies indicate that the m^6^A level in blood/serum reflects the abnormal RNA methylation in the body, which may have potential to be a specific and sensitive biomarker for cancer diagnosis. Total m^6^A levels in blood samples, m^6^A‐related RNAs and m^6^A modifiers can be associated with tumor development and may constitute promising approaches in cancer prognosis.

### 10.2 m^6^A as targets for cancer therapy

#### 10.2.1 Targeting m^6^A‐associated regulators

In the past decades, small molecule chemicals were the most explored as inhibitors to target m^6^A‐related proteins. As the first identified demethylase, inhibitors for FTO were most studied. Over ten FTO‐targeted small molecule inhibitors were developed against cancers, such as Rhein [288], meclofenamic acid [289], quercetin [290], entacapone [291], FB23 and FB23‐2 [292]. We recently developed two FTO inhibitors named 18077 and 18097, which can significantly suppress *in vivo* growth and lung colonization of BC cells [293]. Regarding FTO, inhibitors targeting other m^6^A‐related enzymes were being explored. For example, Yankova et al. [294] described that a catalytic inhibitor of METTL3, named STM2457, could be a potential therapeutic drug against AML due to its oral activity. Sabnis et al. [295] developed new compounds as ALKBH5 inhibitors (IC_50_ = 0.84 μm) for cancer treatment. In addition, a number of natural inhibitors are being discovered continuously, including quercetin for METTL3 [296], betaine for METTL14 [297], clausine for FTO [298], curcumin for ALKBH5 [299] and fusaric acid for YTHs [300, 301]. A list of candidate compounds targeting m^6^A regulators for cancer therapy is presented in Table 3.

**Table 3:** Candidate compounds targeting m^6^A regulators for cancer therapy.

Target	Compound	IC_50_ (μm)	Functions
METTL3	Adenosine 2	8.7	METTL3 inhibitor [302]
METTL3	UZH1a	7	METTL3 inhibitor, reduces the m^6^A/A ratio in mRNAs of three AML cell lines [303]
METTL3	STM2457	0.0169	METTL3 inhibitor, reduces AML growth and increases differentiation and apoptosis [294]
FTO	Rhein	21	FTO inhibitor, exhibits good inhibitory activity on m^6^A demethylation inside cells [288]
FTO	MO‐I‐500	8.7	FTO inhibitor, shows anti‐convulsant activity [304]
FTO	Meclofenamic acid	8	FTO inhibitor [289]
FTO	CHTB	39.24	FTO inhibitor [305]
FTO	R‐2HG	133.3	FTO inhibitor, exerts a broad anti‐leukemic activity *in vitro* and *in vivo* [166]
FTO	FB23‐2	2.6	FTO inhibitor, suppresses proliferation and promotes the differentiation/apoptosis of human AML cell lines [292]
FTO	Entacapone	3.5	FTO inhibitor, mediates metabolic regulation through FOXO1 [291]
FTO	CS1	0.14	FTO inhibitor, suppresses cancer stem cell maintenance and immune evasion [306]
FTO	CS2	2.6	FTO inhibitor, suppresses cancer stem cell maintenance and immune evasion [306]
FTO	Saikosaponin‐d	0.46	FTO inhibitor, shows a broadly suppressed AML cell proliferation and promoted apoptosis and cell‐cycle arrest both *in vitro* and *in vivo* [307]
FTO	Dac51	0.4	FTO inhibitor, blocks FTO‐mediated immune evasion, and synergizes with checkpoint blockade for better tumor control [308]
FTO	FTO‐4	3.4	FTO inhibitor, prevents neurosphere formation in patient‐derived GBM stem cells [309]
FTO	18097	0.64	FTO inhibitor, shows anti‐cancer activities both *in vitro* and *in vivo* [310]
ALKBH5	MV1035	/	ALKBH5 inhibitor, shows an inhibitory effect on GBM [311]
ALKBH5	ALK‐04	/	ALKBH5 inhibitor, enhances the efficacy of cancer immunotherapy [244]
ALKBH5	2‐[(1‐hydroxy‐2‐oxo‐2‐phenylethyl)sulfanyl]acetic acid	0.84	ALKBH5 inhibitor, suppresses cell proliferation at low micromolar concentrations in AML [312]
ALKBH5	4‐[(furan‐2‐yl)methyl]amino‐1,2‐diazinane‐3,6‐dione	1.79	ALKBH5 inhibitor, suppresses cell proliferation at low micromolar concentrations in AML [312]
ALKBH5	Compound 20m	0.021	ALKBH5 inhibitor [313]
IGF2BP1	BTYNB	5	IGF2BP1 inhibitor, targes c‐Myc and inhibits melanoma and ovarian cancer cell proliferation [314]
IGF2BP1	7773	30.45	IGF2BP1 inhibitor, represses Kras and a pro‐oncogenic phenotype in LUAD [315]
IGF2BP2	Benzamidobenzoic acid class and ureidothiophene clas	/	IGF2BP2 inhibitors, show anti‐cancer activities both *in vitro* and *in vivo* [316]

Targeting the expression of m^6^A‐related proteins is another strategy for cancer therapy. RNA interference and CRISPR/Cas9 are techniques that target m^6^A‐related proteins to suppress their expression. The CRISPR system can also be used to identify potential targets that modulate the expression of m^6^A‐related proteins through a genome‐wide CRISPR screen [317].

#### 10.2.2 Single‐site editing of m^6^A‐modified RNAs

Given specific m^6^A modifications on particular RNA molecules can have different effects, modulating single‐site m^6^A on transcript targets may affect the expression of target genes such as oncogenes. We have developed a PspCas13b‐ALKBH5‐based tool named dm^6^ACRISPR for the targeted demethylation of specific mRNAs [318]. Targeting m^6^A modifications of oncogenes such as *EGFR* and *MYC* can significantly suppress their expression and the proliferation of cancer cells [318]; demethylating metabolic gene *PDK4* can reduce its expression and glycolysis of cancer cells [25]. Similarly, Qian's lab has devised an RNA‐targeting‐dCas9 system for site‐specific methylation or demethylation via fusion with a truncated METTL3‐METTL14 heterodimer or full‐length ALKBH5/FTO, respectively [319]. The m^6^A site‐specific manipulation has been summarized recently [320]. The discovery of more potent Cas derivatives, such as Cas13bt, Cas13X, Cas13Y and ABE8, will further improve the CRISPR‐based RNA editing systems and have great potential for applications in various genetic diseases including cancers [320].

Since the oncogenic roles of m^6^A modification have been identified in various types of cancers, studies investigating the potential roles of m^6^A as biomarkers for cancer diagnosis have been performed. In general, levels of total m^6^A, m^6^A‐related RNAs and m^6^A regulators can be used as diagnostic biomarkers for multiple cancers. The relationship between m^6^A/m^6^A‐related markers and cancer progression is satisfactory. Nevertheless, combining m^6^A and clinical used biomarkers can further increase the diagnostic sensitivity of cancer [142], showing a potential application of m^6^A in cancer diagnosis. In addition to the application in diagnosis, targeting m^6^A may serve as a novel direction for cancer therapy due to its effect on tumorigenesis. Nowadays, therapeutic strategies targeting m^6^A mainly include inhibition of enzyme activity and/or expression, and targeted inhibition based on m^6^A editing of specific RNAs. Both *in vitro* and *in vivo* trials show satisfactory results of cancer cell inhibition via either inhibitors or single‐site editing tools. It suggests that targeting m^6^A is a potential and powerful approach for cancer therapy.

## 11 Challenges and perspectives

m^6^A modification is widely distributed in almost all RNA species and has a far‐reaching biological impact. Increasing evidence shows that m^6^A has important regulatory roles in the process of tumorigenesis and cancer development, which can be achieved by the changes in m^6^A‐related protein expression, reader protein activity or the biological functions of m^6^A related‐mRNA and/or ncRNAs. As a matter of fact, m^6^A is expected to become a potential biomarker for cancer diagnosis by monitoring overall m^6^A, m^6^A‐related RNAs and m^6^A modifiers. Since total m^6^A in peripheral blood shows great potential as a biomarker for gastric [143], lung [145] and colorectal [146] cancers, its specific roles in cancer diagnosis warrant further investigation. Moreover, whether m^6^A can be used as a biomarker to distinguish the early stage of cancer patients and healthy people, and whether the levels of m^6^A can be used as a biomarker for prediction or monitoring therapy efficiency remains unclear. In addition, it is reasonable to hypothesize that m^6^A‐methylated transcripts such as mRNAs, ncRNAs and even the RNA fragments may be associated with tumorigenesis and cancer development [321]. However, the potential roles of specific m^6^A‐methylated transcripts in cancer diagnosis need further investigation.

Targeting regulators of DNA and histone methylation have been proven as clinically applicable and important therapeutic strategies [322]. Increasing evidence shows that RNA methylation is a new target for cancer therapy. Developing inhibitors/activators of m^6^A‐related proteins has become a hot spot in the field of anti‐cancer epigenetic drugs. At present, the small molecule candidate drug STM2457 targeting METTL3 is expected to enter the clinical trial stage, which has a significant possibility to become the first RNA epigenetic drug for cancer therapy. However, whether the global methylation/demethylation effect induced by inhibitors/activators of m^6^A‐related proteins would cause unexpected side effects or toxic effects remains up to further investigation. In addition to global demethylation, m^6^A site‐specific editing to target‐specific RNA has gradually become a novel direction of cancer treatment. Similar to CRISPR/Cas9 system targeting DNA, CRISPR proteins targeting RNA (such as Cas13b, CasRx) combined with m^6^A‐related proteins can achieve site‐specific deposition and demethylation of m^6^A, leading to the degradation, translation and other effects of specific targets [319]. Compared with CRISPR/Cas9, CRISPR targeting RNA does not affect the DNA, which can circumvent mutations caused by off‐target effects being passed down to the next generation. Therefore, a site‐targeting m^6^A‐editing method would be a promising direction for tumor treatment. Remarkably, numerous challenges need to be overcome before the clinical application of a targeted m^6^A‐editing method, such as ways to achieve sufficient delivery *in vivo*, approaches to target tumor cells specifically, means to reduce off‐target effects, and more. An in‐depth study of m^6^A distribution, functions and biological impact will broaden our understanding of RNA epigenetic regulation of tumor development. We therefore believe that an increasing number of novel, specific, effective and promising methods targeting m^6^A modifications could be developed, being a new direction for both cancer diagnosis and targeted therapy.

## Conflict of interest

The authors declare no conflict of interest.

## Author contributions

Conception and design: JL, HW. Writing, review and/or revision of the manuscript: ZW, JZ, JL, HW. Collation of information: HZ, LG.

## Data Availability

Data openly available in a public repository.
